# Reduced lateralization of the language network in the blind and its relationship with white matter tract neuroanatomy

**DOI:** 10.3389/fnhum.2024.1407557

**Published:** 2024-08-12

**Authors:** Gabriela Dzięgiel-Fivet, Katarzyna Jednoróg

**Affiliations:** Laboratory of Language Neurobiology, Nencki Institute of Experimental Biology, Polish Academy of Sciences, Warszaw, Poland

**Keywords:** lateralization, corpus callosum, blind, reading, speech-processing, white matter, fMRI, FA

## Abstract

Several previous studies reported reduced leftward lateralization in blind participants’ samples compared to the sighted population. The origins of this difference remain unknown. Here, we tested whether functional lateralization is connected with the structural characteristics of white matter tracts [corpus callosum (CC), uncinate fasciculus (UF), and superior longitudinal fasciculus (SLF)], as suggested by previous studies conducted in the typical sighted population. Twenty-three blind and 21 sighted adult participants were tested during fMRI with a semantic decision paradigm presented both auditorily and in the modality appropriate for reading (tactually for the blind and visually for the sighted). Lateralization indices (LI) were calculated based on the activations. The fractional anisotropy (FA) measure was extracted from the white matter tracts of interest. Correlation analyses testing the relationship between FA and LI were conducted. The reduced leftward lateralization of both speech processing and reading-related activations was replicated. Nevertheless, the relationship between the structural integrity of the CC and LI and between the asymmetry of the intrahemispheric tracts and LI was not confirmed, possibly due to the lack of power. The sources of the reduced lateralization of the language network in the sensory-deprived population remain unknown. Further studies should account for environmental variables (e.g., the frequency of contact with written language) and the complexity of the factors that may influence the functional lateralization of the human brain.

## 1 Introduction

Despite visual deprivation, neural networks processing spoken and written language exhibit similarities between blind and sighted populations (Sadato et al., 1996; Büchel et al., 1998a,b; Burton et al., 2002a,b; Büchel, 2003; Bedny et al., 2011, 2015). Although the most striking difference is the involvement of the occipital cortex in language processing in the blind, other discrepancies have also been observed (Dzięgiel-Fivet et al., 2021). Reduced leftward lateralization of language-related activations is reported in the blind compared to the sighted (Lane et al., 2017; Tian et al., 2022). Lane et al. (2017) compared LI for various spoken language processing tasks (all focused on syntactic processing), finding significantly reduced lateralization in blind individuals. This finding was replicated with a simpler linguistic task (single-word perception with a memory probe to increase attention) by Tian et al. (2022), showing reduced leftward lateralization for reading and speech processing in the blind.

The reasons for the decreased lateralization of the language network in the blind are not fully understood. It was suggested that the spatial nature of Braille processing decreases the left hemisphere dominance in language processing in the blind, as it is the right hemisphere that is specialized in spatial processing (Karavatos et al., 1984). However, more recent accounts propose that the lateralization of the reading-related activations is predicted by the lateralization of the response to spoken language in both the blind (Tian et al., 2022) and sighted (Cai et al., 2010; Van der Haegen et al., 2012). Thus, the factors influencing the laterality of speech processing seem decisive for the reading network lateralization.

Differences between the blind and the sighted have also been observed for brain structure. Several diffusion imaging studies have shown changes in FA, a measure of white matter tract integrity (Beaulieu, 2002), in blind subjects, particularly in visual pathways (Ptito et al., 2008), and the CC (Reislev et al., 2016; Anurova et al., 2019). In the sighted, links between the white matter structures and functional lateralization were suggested. However, the correlation between the characteristics of white matter structures and the lateralization of language-related activations has not been studied in the blind.

Here, we focus on two structural measures that were previously shown to be connected to functional lateralization in the sighted. First, the CC—the largest white matter structure connecting the two hemispheres—has been found to be important for functional lateralization (Josse et al., 2008; Hinkley et al., 2016; Karolis et al., 2019). Two competing hypotheses agree that the structural characteristics of the CC play an important role in functional lateralization (Clarke et al., 1993; Ringo et al., 1994). The first one proposes that the role of the CC is inhibitory (Clarke et al., 1993). Homologous structures in the two hemispheres inhibit each other using the callosal connections. Thus, greater efficiency of the callosal connections would lead to increased lateralization. In patients with CC agenesis, language lateralization was decreased or non-existent (Hinkley et al., 2016). Moreover, in the neurotypical population, the CC volume was positively correlated to the degree of left-lateralization of activations in linguistic tasks (Josse et al., 2008). The second hypothesis proposes that lateralization is connected to the increase in the size of the brain, limiting the delays caused by the interhemispheric transfer (Ringo et al., 1994). Thus, lateralization would decrease with increased interhemispheric connections (i.e., a negative correlation). A recent, large-sample study supports this hypothesis (Karolis et al., 2019), showing a relationship between functional lateralization and diffusion-based connectivity measures.

Second, the asymmetry of intrahemispheric tracts, implicated in language processing, is considered in the current study. Structures such as arcuate fasciculus and UF were found to be asymmetrical in terms of white matter integrity (represented by FA) (Büchel et al., 2004; Ocklenburg et al., 2013). Moreover, their asymmetry was correlated with measures of functional lateralization (Ocklenburg et al., 2013, 2016).

The study aimed to explore if white matter changes in blind individuals relate to reduced lateralization in reading and spoken word processing. A correlation between CC structural characteristics and language lateralization and the asymmetry of intrahemispheric tracts and lateralization, akin to sighted individuals, would imply that the mechanisms governing the functional lateralization of language (or at least some of them) are independent of sensory deprivation and changed reading modality. We anticipated a significant correlation in both groups. A positive correlation would support the hypothesis regarding the inhibitory role of CC (Clarke et al., 1993). Conversely, a negative correlation would support the hypothesis suggesting a decrease in lateralization with increased interhemispheric connections (Ringo et al., 1994). Previous literature presented arguments for both hypotheses. However, the studies supporting the second one (Karolis et al., 2019; Yang et al., 2022) are more recent and conducted on much larger samples, rendering the second hypothesis more probable. Additionally, we sought to replicate prior findings of reduced leftward lateralization in blind individuals compared to sighted ones using a semantic decision task. Replicating this decreased lateralization with a different linguistic task would bolster the generalizability of the effect, shedding light on the consistent differences in neural correlates of language processing between blind and sighted individuals.

## 2 Methods

### 2.1 Participants

Data for this study were gathered from two projects. First, diffusion-weighted imaging (DWI) was conducted on 25 blind and 25 sighted adults who were matched in age [blind = 36.1 (SD = 10.3); sighted = 35.4 (SD = 9.89)], sex (16 female per group), handedness (24 right-handed, 1 left-handed per group—evaluated using the Polish version of the Edinburgh Handedness Questionnaire, Dragovic, 2004), and education. Two years later, these participants underwent fMRI scans. Twenty-three blind (mean age: 39.3 (SD = 10.6), 15 females, 22 right-handed) and 21 sighted (mean age: 37.6 (SD = 8.28), 13 females, 20 right-handed) individuals participated in the second session. Two blind subjects were excluded from the reading condition due to excessive movement. Laterality analyses were repeated on right-handed participants only, yielding similar results (see Supplementary Materials). All visually impaired participants lost sight before age 3 and had no neurological or developmental disorders. Twelve out of 25 blind subjects (11 out of whom participated in the second session) reported some residual vision—light or movement perception at most. All of them were declared legally blind. Detailed information on the participants’ demographics is available in an online repository, with the rest of the published data.

The blind group was a convenience sample—all volunteers matching the inclusion criteria (early blindness—blindness before the onset of Braille reading acquisition, Braille as primary script for reading acquisition, no knowledge of the print Latin alphabet) were tested. The sighted participants were chosen to ensure matching to the blind group in terms of age, sex, education level, and handedness.

The study was approved by the ethics committee at the Psychology Department of the University of Warsaw. The studies were conducted in accordance with local legislation and institutional requirements. The participants provided their written informed consent to participate in this study. The consent form was beforehand presented to the blind participants in a screen reader-friendly format.

### 2.2 DWI acquisition and analysis

The data were acquired using a 3 T Siemens Trio Scanner with a 12-channel head coil. An echo-planar imaging (EPI) sequence was used for DWI with 64 diffusion directions (b = 1,300 s/mm^2^), including one non-diffusion-weighted image (b = 0 s/mm^2^) with AP phase encoding. Each volume comprised 64 axial slices (slice thickness = 2 mm, TR = 8,700 ms, TE = 92 ms, FOV = 256 × 256 mm, matrix size = 128 × 128).

FSL (Smith et al., 2004) was used for DWI data analysis, following standard preprocessing. Brain masks were created using BET, eddy currents, and motion were corrected, and intensity outliers were replaced using -repol. Quality control checks were performed using QUAD and SQUAD, with no exclusions due to data homogeneity (no absolute or relative motion outliers, nor CNR outliers above 3SD). Voxel-wise statistical analysis of FA data was conducted using tract-based spatial statistics (TBSS). FA data were aligned using FNIRT and then transformed into 1 × 1 × 1 mm MNI152 space through alignment to the transformed target subject (selected automatically). Afterward, the FA data were projected onto a mean FA skeleton. Voxel-wise cross-subject statistics were performed using randomise. Similar analyses were conducted on mean diffusivity (MD) (see Supplementary Materials for results).

### 2.3 fMRI task and scanning procedure

A semantic decision task was used to elicit language-related activations, contrasted with a perceptual decision task as a control. Both tasks were completed audibly by both blind and sighted subjects, visually by sighted individuals, or tactually by the blind. Each fMRI run included one modality for presenting both tasks. There were two runs per modality, totaling four runs per subject. In the semantic task, subjects distinguished animate from inanimate objects, responding accordingly. The stimuli were short (3–5 letter) words representing common objects. The auditory control task involved discerning additional tones in noise bursts. In visual and tactile control tasks, subjects identified spaces in non-linguistic stimulus strings (hash strings for the visual condition and six-dot signs for the tactile condition). For task performance results, see Supplementary Materials.

Tasks followed a block paradigm with 20 blocks per run—10 for semantic and 10 for control tasks. Each block comprised four trials with stimulus presentation and response time. Blocks were separated by intervals lasting 3 to 6 s (4.5 s on average). Blocks began with an auditory cue, followed by a brief pause (2000 ms between the cue and the first stimulus). Auditory and visual stimuli were shown for 1,000 ms, while tactile stimuli lasted 2,500 ms to match the slower reading tempo. Subjects had 1,500 ms to respond after each stimulus, resulting in block durations of 10 s for visual and auditory and 16 s for tactile.

Scanning commenced with anatomical scans, followed by functional scans. Before each modality’s task run, a brief training session was conducted, comprising four blocks each of control and semantic tasks with stimuli different from experimental sessions. The order of runs was counterbalanced across participants.

### 2.4 fMRI acquisition and analysis

The data were gathered using a 3 T Siemens Trio Scanner with a 12-channel head coil. Functional images were acquired via a whole-brain EPI sequence, comprising 35 slices with 3.5 mm slice thickness, TR = 2000 ms, TE = 30 ms, flip angle = 80°, FOV = 192 mm, matrix size = 64 × 64, and voxel size: 3 × 3 × 3.5 mm. Anatomical images were acquired using a T1-weighted MPRAGE sequence, with 176 slices, 1 mm slice thickness, TR = 2,530 ms, TE = 3.32 ms, flip angle = 7°, matrix size = 256 × 256, and voxel size = 1 × 1 × 1 mm.

SPM12 (Wellcome Trust Centre for Neuroimaging, London, UK) running on Matlab2017b (The MathWorks Inc., Natick, MA, United States) was used for MRI data preprocessing and whole-brain analyses. Standard preprocessing included realignment (to the mean functional image, estimated before but resliced after slice-time correction), slice-time correction, coregistration (to the SPM template), segmentation, normalization to 2 × 2 × 2 mm MNI space, and smoothing (7 mm isotropic Gaussian kernel). The ART toolbox was used to create movement regressors and detect excessive in-scanner motion (the default: movement over 1 mm and rotation over 0.2 radians in relation to the previous volume and intensity differing over 3 SD from the mean global image intensity). Participants with less than 80% artifact-free volumes were excluded.

Voxel-wise GLM analysis incorporated condition blocks (semantic or control) and auditory cues, convolved with the canonical hemodynamic function. Motion regressors were added to the model. Contrasts comparing semantic to control tasks delineated speech and reading-related activations, thresholded at *p* < 0.001 unc., with cluster-level FWE threshold at *p* < 0.05. Anatomical structures were labeled using the “atlasreader” function based on the AAL2 atlas.

### 2.5 Lateralization index extraction

LI were computed using the LI toolbox (Wilke and Schmithorst, 2006), using bootstrap thresholding while excluding a 10 mm area around the midline. LI calculation focused on relevant brain regions, utilizing masks in bilateral triangular inferior frontal and middle temporal areas, and ventral occipitotemporal cortex (vOT). Frontal and temporal masks were selected from a publicly available set of language ROIs^1^ similar to previous studies (Lane et al., 2017; Tian et al., 2022). The ventral occipital mask was created by intersecting spheres around peak coordinates from Lerma-Usabiaga et al. (2018) and Kim et al. (2017) with ITG and FG masks from the AAL3 atlas. These were flipped to the right hemisphere for bilateral masking using the MarsBaR toolbox (Brett et al., 2002).

LIs were extracted separately from the three ROIs (inferior frontal, middle temporal, and ventral occipitotemporal) for group difference analysis and from the combined ROI mask for correlation analysis.

## 3 Results

### 3.1 Comparison of the integrity of white matter tracts between blind and sighted

**Figure 1:**
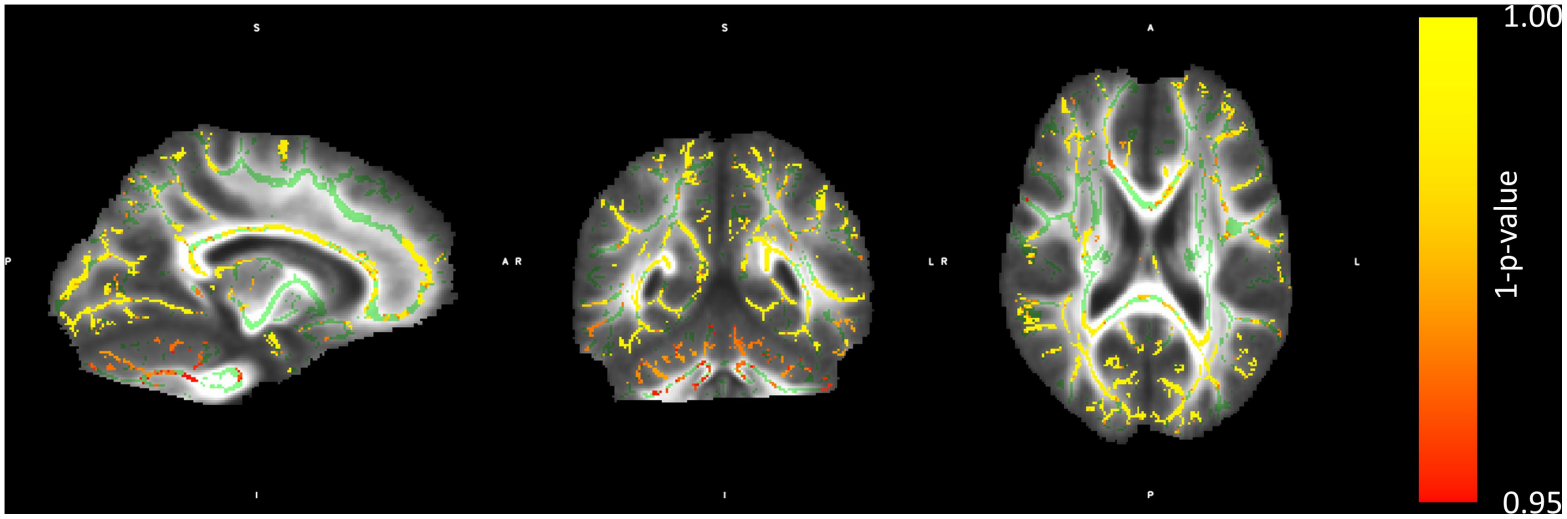
Regions with lower FA in the blind than in the sighted subjects. The color bar represents a metric of 1–*p*-value of the permutation group comparison statistics. Structures marked in green represent the mean FA skeleton.

The group-wise comparison was conducted using permutation testing implemented in randomise, applying threshold-free cluster enhancement (TFCE). The blind group showed reduced FA in numerous white matter tracts compared to sighted controls. Importantly, significant differences were found in the CC (genu, body, and splenium), bilateral superior longitudinal fasciculi, and white matter tracts related to visual processing in the typical population (optic radiation, see Figure 1). Sighted subjects did not show any areas of reduced FA in comparison to the blind subjects.

The mean FA was extracted from the body, genu, and splenium of the CC, SLF, and UF. The ROIs were defined based on the JHU ICBM 81 White-Matter Labels Atlas accessible in FSL. In the case of the SLF and UF ROIs, it is the asymmetry of these structures that is considered important for language lateralization (Ocklenburg et al., 2016); thus, the difference between the left and right hemisphere was considered (LH_FA—RH_FA). The descriptive statistics of the FA values within groups for the 5 ROIs are presented in Table 1.

**Table 1:** FA values within ROIs for the blind and sighted participants.

		Blind	Sighted
CC body	Mean	0.71	0.73
	SD	0.06	0.02
	*U*	172	
	*p*	0.006	
	*r*	0.39	
CC genu	Mean	0.71	0.73
	SD	0.06	0.02
	*U*	238	
	*p*	0.152	
	*r*	0.2	
CC Splenium	Mean	0.76	0.78
	SD	0.03	0.02
	*U*	138	
	*p*	< 0.001	
	*r*	0.48	
SLF (L-R)	Mean	0	0
	SD	0.02	0.01
	*U*	322	
	*p*	0.863	
	*r*	0.03	
UF (L-R)	Mean	0	−0.01
	SD	0.07	0.04
	*U*	364	
	*p*	0.325	
	*r*	0.14	

SD–standard deviation, U–Mann–Whitney *U*-test statistic, *p*–*p*-value, *r*–effect size measure.

As the distribution of the FA values was not always normal within the group, Mann–Whitney U-test was used to compare the group. Significant differences between the groups were found for CC body and CC splenium, with sighted participants presenting higher FA values than the blind participants.

### 3.2 fMRI group-level activations

**Figure 2:**
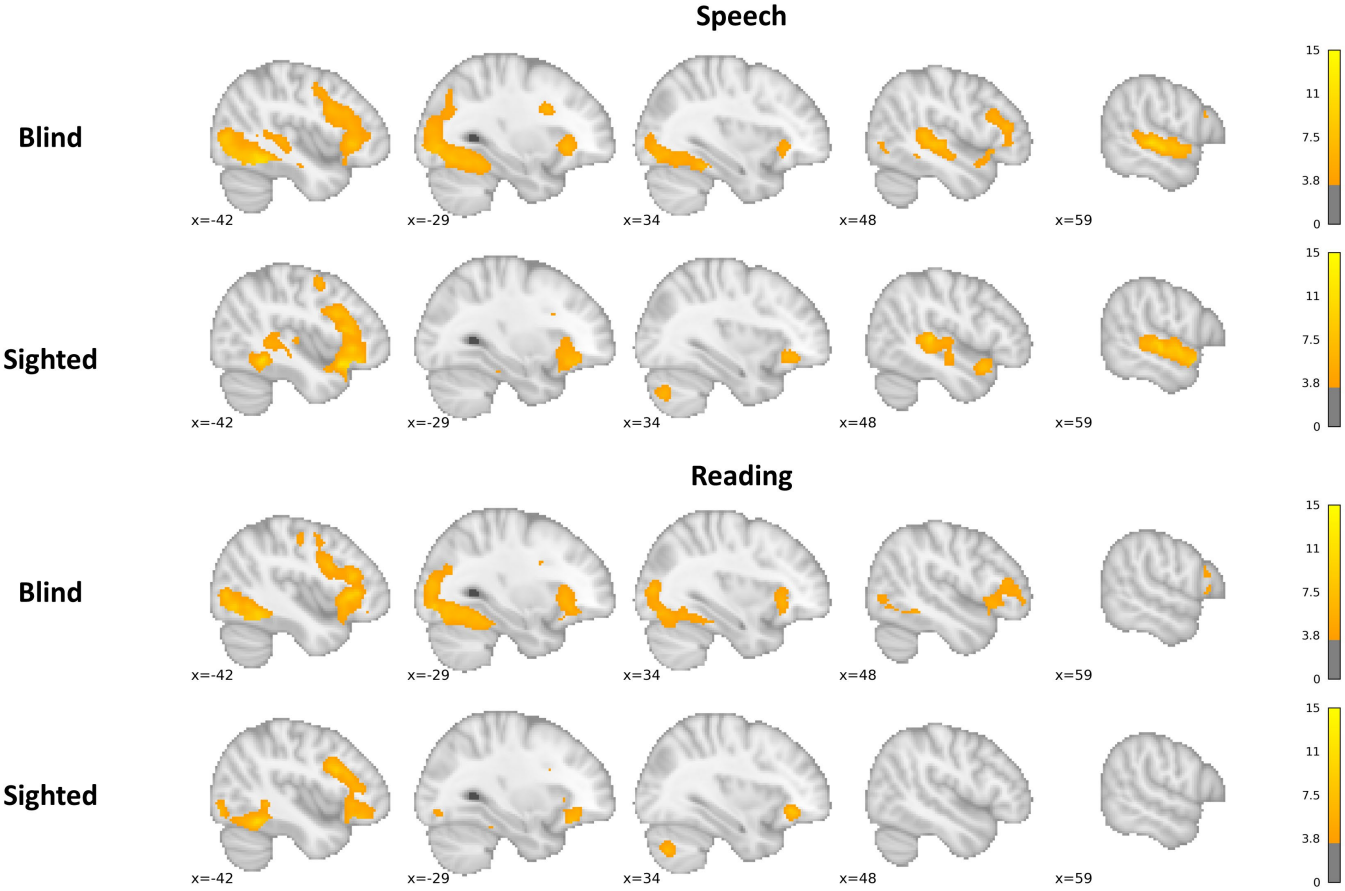
Activations during speech processing and reading in the blind and sighted groups. The color bar represents *t*-values.

Speech processing evoked extensive and bilateral perisylvian activations in both sighted and blind subjects (Figure 2; Supplementary Table S2). Blind subjects activated the occipital cortex, including V1 and the ventral occipital cortex bilaterally, more than the sighted subjects. Additionally, clusters in the right IFG and left SPL were also activated to a greater extent by the blind subjects. There was no significant cortical activation greater in the sighted group than in the blind group.

Reading evoked bilateral vOT and IFG activation in the blind group. In the sighted, these activations were localized only in the left hemisphere, with only one cerebellum cluster in the right hemisphere (Figure 2; Supplementary Table S3). The blind group activated the bilateral occipital cortex, including V1 and vOT, more than the sighted group, as well as the right IFG, supplementary motor cortex, and left precentral/postcentral gyri cluster. The sighted group did not present any significant cortical activation above the activations present in the blind group.

### 3.3 Comparison of the lateralization of language processing between blind and sighted

Lateralization of language processing was assessed in three language network regions of interest (ROIs): triangular inferior frontal cortex (IF Tri), middle temporal cortex (Temp Mid), and vOT, separately for speech and reading contrasts. A three-way mixed ANOVA was conducted with a group (blind vs. sighted) as a between-subjects factor and ROI and condition as within-subjects factors.

Significant main effects were found for group (*F*(1, 37) = 7.17, *p* = 0.011, η_p_^2^ = 0.16) and ROI (*F*(2, 74) = 12.11, *p* < 0.001, η_p_^2^ = 0.25). The condition-by-ROI interaction was marginally significant (*F*(2,74) = 2.74, *p* = 0.071, η_p_^2^ = 0.07), reaching significance when only right-handed participants were included (see Supplementary Materials). Other interactions and main effects were not significant (main effect of condition: *F*(1, 37) = 0.19, *p* = 0.668; group by condition interaction: *F*(1, 37) = 1.44, *p* = 0.238; group by ROI interaction: *F*(2, 74) = 0.81, *p* = 0.450; and group by ROI by condition interaction: *F*(2, 74) = 0.99, *p* = 0.476).

**Figure 3:**
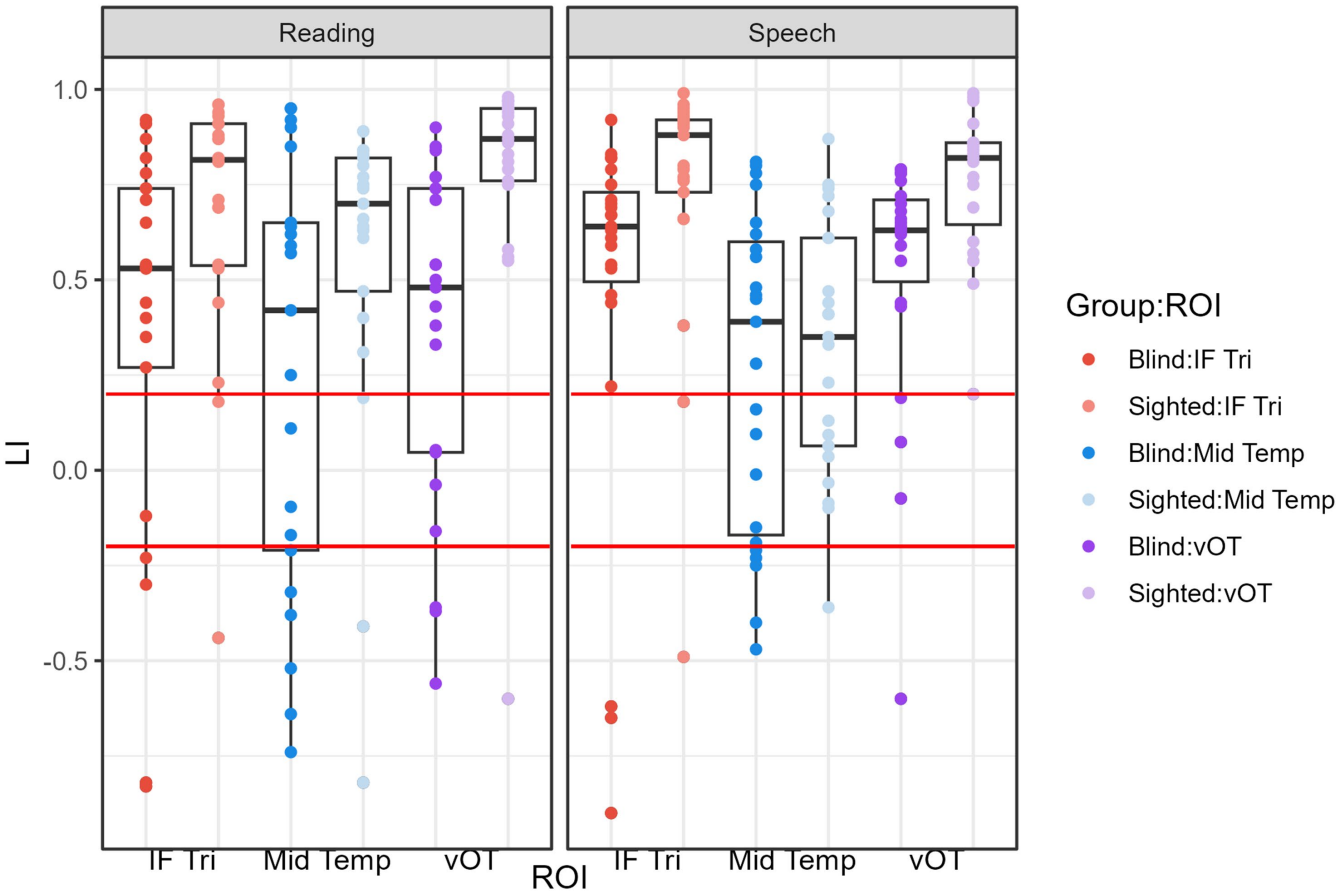
LI values within groups and ROIs for the reading and speech processing conditions. The red lines mark the values of LI = 0.2 and LI = −0.2. Values greater than LI = 0.2 indicate leftward lateralization and values lower than LI = −0.2 indicate rightward lateralization.

The condition-by-ROI interaction revealed significant ROI effects in both conditions for the sighted group but only for the speech condition in the blind group. The temporal ROI generally showed lower LI values compared to frontal and vOT ROIs. However, in the reading condition for the blind group, all ROIs exhibited similar lateralization (see Figure 3).

The main effect of the group indicated that sighted subjects had higher LI values on average than the blind group, indicating stronger leftward lateralization in this group.

Nevertheless, even in the blind group, the majority of participants presented leftward lateralization (LI greater than 0.2; see Table 2, Figure 3) for both tasks and all of the ROIs.

**Table 2:** Number of participants with given lateralization by ROI.

	Blind						Sighted					
	Reading			Speech			Reading			Speech		
	L	R	Blilateral	L	R	Blilateral	L	R	Blilateral	L	R	Blilateral
IF Tri	16	4	1	20	3	0	18	1	1	19	1	1
Temp mid	12	6	3	13	5	5	18	2	1	13	1	7
vOT	14	3	4	19	1	3	20	1	0	19	0	0

### 3.4 Correlations between the LI and white matter tract characteristics

As we hypothesized that in both groups the relationship between white matter integrity and lateralization would be the same, the correlation analysis included both groups. Partial Spearman correlations controlling for visual impairment status (blind vs. sighted) were computed between LI from the global language mask (sum of three ROIs) and structural measures: FA of CC parts (body, genu, splenium) and SLF and UF asymmetry (left FA minus right FA). None of the correlations reached significance (Table 3); the results were corrected using the Bonferroni–Holm correction for multiple comparisons. To confirm consistency, correlations were computed within groups, and then differences between groups were assessed (using a bootstrap approach implemented in the *bootcorci* package). No significant differences were found, indicating similar relationships between LI and FA measures across blind and sighted groups. Similar results were obtained for correlations between LI and MD measures (see Supplementary Table S5).

**Table 3:** Spearman correlation between FA and lateralization of the language network.

		Reading: all	Speech: all	Reading: blind	Speech: blind	Reading: sighted	Speech: sighted
CC Body	*rho*	−0.07	−0.09	0.21	0.14	−0.31	−0.51
	*p*	0.648	1.000	1.000	1.000	0.747	0.104
CC Genu	*rho*	0.13	−0.36	0.05	−0.24	0.03	**−0.61**
	*p*	1.000	0.081	1.000	1.000	0.910	**0.026**
CC Splenium	*rho*	0.10	−0.04	0.22	0.10	−0.44	−0.49
	*p*	1.000	1.000	1.000	1.000	0.271	0.102
SLF asymmetry	*rho*	0.85	0.02	0.13	−0.17	−0.21	0.21
	*p*	0.740	0.915	1.000	1.000	1.000	0.798
UF asymmtery	*rho*	−0.26	−0.07	−0.02	−0.05	−0.14	0.04
	*p*	0.440	1.000	0.931	0.832	1.000	0.864

The correlations for all participants are partial correlation controlled for the blind status. Results surviving Holm–Bonferroni correction are highlighted in bold.

## 4 Discussion

The main objective of this study was to test for the correlation between the characteristics of white matter structures (the integrity of different parts of the CC and the asymmetry of intrahemispheric white matter structures) and functional lateralization of language in the visually deprived population. Our analyses do not confirm such a correlation. The second objective of the study, the replication of the results showing a decreased lateralization of language-related activations in the blind compared to the sighted, was attained. In the blind participants, activations evoked by both reading and speech processing were less lateralized than in the sighted participants. Lateralization of the language-related activations was not dependent on the reading hand used by blind participants (see Supplementary Materials), as suggested before (Karavatos et al., 1984).

What is worth noting is that the variability of the LI seems greater in the blind than in the sighted group, especially when the reading-related activations are considered (see Figure 3). This may be an effect of greater variability of the reading acquisition experience, as well as the diverse amount of contact with written language in the blind compared to the sighted. Some blind participants reported using Braille daily, while some said that since graduating from school, they had little contact with written language. Unfortunately, we did not directly measure the characteristics of reading acquisition (apart from the moment of the beginning of learning, which was similar for all of the participants—around the age of 7 which is the age of obligatory schooling), nor the everyday Braille exposure. Future research should take these factors into account.

The null results of the correlation analyses may stem from several factors. First, although our sample size was similar to other studies conducted with blind participants (Lane et al., 2017; Anurova et al., 2019; Tian et al., 2022), our study may have been underpowered. We estimated correlations between the white matter characteristics and LI in the blind and the sighted groups separately (Table 3, for MD, see Supplementary Table S3). In the sighted, only the correlation between the FA (and MD) of the genu of the CC and the LI of speech processing survived the multiple comparison correction. Thus, we were not able to convincingly replicate previous findings showing the correlations between the structural characteristics of the CC and lateralization (Josse et al., 2008; Karolis et al., 2019) nor between the asymmetry of the intrahemispheric tracts and lateralization (Ocklenburg et al., 2016) even in the sighted group. The only significant correlation was negative, supporting the hypothesis on the role of the CC in increasing the hemispheric transfer of information and decreasing lateralization (Ringo et al., 1994). The insignificant correlations with other parts of the CC in general were in the same direction. Interestingly in the blind sample, the correlations were rather positive, supporting the hypothesis on the inhibitory role of CC (Clarke et al., 1993). Nevertheless, the correlations were not significant and thus they cannot be interpreted in a conclusive way.

Second, we focused on the white matter integrity, while many studies analyzing the structure of the CC used mainly volume and surface measures (Hines et al., 1992; Yazgan et al., 1995; Josse et al., 2008). The recent large-scale study supporting the hypothesis of the CC’s role in increasing the interhemispheric transfer and thus decreasing lateralization used diffusion-based measures of axonal water fraction (Karolis et al., 2019) or fiber streamlines (Yang et al., 2022). Here, the measure of FA (with additional analyses of MD, as suggested by Figley et al., 2022) was used as it may be interpreted as a measure of white matter tract integrity (Beaulieu, 2002). It may nevertheless be more sensitive to different characteristics of white matter tracts than the measures used in the previous studies.

Another reason that may have contributed to the null results is the fact that lateralization is a phenomenon influenced by multiple factors that contribute only moderately or weakly to hemispheric dominance. Several hypotheses connecting structural characteristics of the brain and lateralization have been proposed. None of them is strongly supported by the literature as a unique factor determining functional lateralization. This is why a triadic model was proposed by Ocklenburg et al. (2016). It underlines the independent importance of gray matter asymmetries, the interaction between hemispheres mediated by the CC, and the asymmetry of intrahemispheric tracts. Independent contributions of these factors, however, seem weak and may require big samples to be demonstrated.

The reasons for the decreased lateralization observed in the visually deprived population, as well as the sources of the lateralized organization of the human brain, remain unknown. This study replicates the findings of decreased laterality of language-related activations in the blind with a different task than the ones used before, increasing the generalizability of these results. Further research is needed to understand the mechanisms governing the functional organization of the brain, as well as their interaction with plasticity provoked by sensory deprivation.

## Data availability statement

The datasets presented in this study can be found in online repositories. The names of the repository/repositories and accession number(s) can be found at: https://osf.io/k56n2/.

## Ethics statement

The study was approved by the ethics committee at the Psychology Department of the University of Warsaw. The studies were conducted in accordance with the local legislation and institutional requirements. The participants provided their written informed consent to participate in this study. The consent form was beforehand presented to the blind participants in a screen reader-friendly format.

## Author contributions

GD-F: Conceptualization, Data curation, Formal analysis, Funding acquisition, Investigation, Methodology, Project administration, Visualization, Writing – original draft, Writing – review & editing. KJ: Conceptualization, Funding acquisition, Supervision, Writing – review & editing.

## Footnotes

^1^https://evlab.mit.edu/funcloc/index.html
