# Supplementary material for: Reduced lateralization of the language network in the blind and its relationship with white matter tract neuroanatomy
Source: Front Hum Neurosci. 2024 Aug 12;18:1407557. doi: 10.3389/fnhum.2024.1407557 (PMC11345183; doi:10.3389/fnhum.2024.1407557)
Supplement: Supplementary file 1 [file Data_Sheet_1.docx]

***Supplementary Material***

1. fMRI task performance

Accuracy in both the auditory and reading tasks was compared between the groups. In both groups, accuracy for both tasks was very high (see Table S1). Mann’s Whitney U test was used, as the distribution of scores within groups sometimes departed from normality. The differences between the groups were insignificant with one exception: Sighted participants performed better in the reading words condition than the blind group.

**Supplementary Table 1**. Descriptive statistics and group comparison of the in-scanner task. Accuracy is in percent correct.

|  | Blind *M (SD)* | Sighted *M (SD)* | Comparison |
| --- | --- | --- | --- |
| Auditory words | 94 (4) | 96 (3) | *U* = 208, *p* = 0.400 |
| Auditory control | 98 (3) | 95 (12) | *U* = 236, *p* = 0.900 |
| Reading words | 95 (5) | 98 (2) | *U* = 139, *p* = 0.020 |
| Reading control | 95 (1) | 96 (1) | *U* = 203, *p* = 0.400 |

**Supplementary Table 2.** Speech-related activations (semantic > control task contrast). The anatomical structures are described according to the AAL2 using the “atlasreader” function, Hem. – hemisphere, x, y, z – peak coordinates, t – peak t-value

| Brain region | Hemisphere | x | y | z | *t* | voxels |
| --- | --- | --- | --- | --- | --- | --- |
| **Blind** |  |  |  |  |  |  |
| Middle Temporal Gyrus, Middle Occipital Gyrus, Inferior Frontal Gyrus (pars triangularis, opercularis, orbitalis), Lingual Gyri, Fusiform Gyri, Calcarine, Superior Occipital Gyrus, Cuneus, Superior Temporal Gyrus, Inferior Occipital Gyri, Cerebelum, Inferior Temporal Gyri, Precentral Gyrus, Insula, Middle Occipital Gyrus, Superior Occipital Gyrus, Superior Temporal Pole, Superior Parietal Lobule, Middle Frontal Gyrus | L/R | -56 | -20 | 0 | 12.48 | 15542 |
| Superior Temporal Gyrus, Inferior Frontal Gyrus (pars triangularis, orbitalis), Middle Temporal Gyrus, Superior Temporal Pole, Insula | R | 58 | -26 | 0 | 10.85 | 2240 |
| Supplementary Motor Area, Middle Cingulate Gyrus, Superior Frontal Gyrus medial | L/R | 8 | 14 | 46 | 7.38 | 668 |
| **Sighted** |  |  |  |  |  |  |
| Inferior Frontal Gyrus (pars triangularis, orbitalis , opercularis), Middle Temporal Gyrus, Superior Temporal Gyrus, Superior Temporal Pole, Inferior Temporal Gyrus, Insula, Posterior Orbital Gyrus, Fusiform Gyrus, Precentral Gyrus | L | -62 | -8 | -4 | 14.8 | 6146 |
| Superior Temporal Gyrus, Middle Temporal Gyrus, Superior Temporal Pole, Heschl Gyrus | R | 60 | -18 | -2 | 10.22 | 1985 |
| Supplementary Motor Area, Superior Frontal Gyrus | L | -4 | 20 | 54 | 7.45 | 1127 |
| Cerebellum | R | 14 | -84 | -30 | 9.69 | 1022 |
| Inferior Frontal Gyrus (pars orbitalis) | R | 32 | 30 | -8 | 6.42 | 223 |
| Precentral Gyrus | L | -40 | 0 | 50 | 6.48 | 147 |
| **Blind > Sighted** | |  |  |  |  |  |
| Middle Occipital Gyrus, Lingual Gyri, Fusiform Gyri, Calcarine, Inferior Occipital Gyri, Superior Occipital Gyrus, Cerebelum Cuneus, Middle Temporal Gyri, Inferior Temporal Gyri | L/R | -38 | -78 | 2 | 8.57 | 8567 |
| Inferior Frontal Gyrus (pars triangularis) | R | 42 | 36 | 14 | 5.15 | 275 |
| Superior Parietal Lobule | L | -22 | -66 | 44 | 4.64 | 194 |
| **Sighted > Blind** | |  |  |  |  |  |
| White matter | L | -30 | -54 | 4 | 5.33 | 178 |

**Supplementary Table 3.** Reading-related activations (semantic > control contrast). The anatomical structures are described according to the AAL2 using the “atlasreader” function, Hem. – hemisphere, x, y, z – peak coordinates, t – peak t-value

| Brain region | Hemisphere | x | y | z | *t* | voxels |
| --- | --- | --- | --- | --- | --- | --- |
| **Blind** |  |  |  |  |  |  |
| Middle Occipital Gyrus, Lingual Gyri, Fusiform Gyri, Calcarine, Superior Occipital Gyrus, Inferior Occipital Gyrus, Middle Occipital Gyrus, Middle Temporal Gyrus, Inferior Temporal Gyrus, Inferior Occipital Gyrus, Cuneus, Inferior Temporal Gyrus, Superior Occipital Gyrus | L/R | -42 | -52 | -14 | 9.29 | 8873 |
| Inferior Frontal Gyrus (pars triangularis, opercularis, orbitalis), Precentral Gyrus, Insula, Postcentral Gyrus, Posterior Orbital Gyrus, Superior Temporal Pole | L | -48 | 30 | 18 | 9.49 | 3232 |
| Supplementary Motor Area, Middle Cingulate Gyrus, Superior Frontal Gyrus | L/R | 8 | 10 | 48 | 7.85 | 1390 |
| Inferior Frontal Gyrus (pars traingularis, orbitalis), Insula | R | 44 | 24 | -6 | 7.19 | 1070 |
| **Sighted** |  |  |  |  |  |  |
| Inferior Frontal Gyrus (pars tirangularis, orbitalis, opercularis), Precentral Gyrus, Posterior Orbital Gyrus, Middle Frontal Gurus, Insula, Anterior Orbital Gyrus, Superior Temporal Pole | L | -52 | 22 | 22 | 9.91 | 2245 |
| Fusiform Gyrus, Inferior Occipital Gyrus, Inferior Temporal Gyrus, Middle Temporal Gyrus | L | -42 | -52 | -18 | 10.37 | 1051 |
| Cerebellum | R | 10 | -82 | -32 | 7.79 | 882 |
| Middle Temporal Gyrus, Superior Temporal Gyrus | L | -54 | -46 | 8 | 5.09 | 425 |
| Inferior Frontal Gyrus (pars orbitalis) | R | 34 | 34 | -8 | 7.97 | 155 |
| **Blind > Sighted** |  |  |  |  |  |  |
| Middle Occipital Gyrus, Lingual Gyri, Fusiform Gyri, Calcarine, Cerebelum, Superior Occipital Gyrus, Inferior Occipital Gyri, Middle Occipital Gyrus, Inferior Temporal Gyri, Cuneus, Superior Occipital Gyrus, Middle Temporal Gyri, Superior Parietal Lobule | L/R | 32 | -82 | 10 | 9.77 | 10515 |
| Supplementary Motor Area, Middle Cingulate Gyrus | L/R | 4 | 6 | 50 | 4.87 | 850 |
| Inferior Frontal Gyrus (pars triangularis, orbitalis), Insula | R | 58 | 22 | 6 | 5.47 | 702 |
| Postcentral Gyrus, Postcentral Gyrus | L | -50 | -6 | 38 | 4.85 | 357 |
| **Sighted > Blind** |  |  |  |  |  |  |
| White matter | L | -34 | -44 | 20 | 4.88 | 775 |
| White matter | R | 16 | -40 | 16 | 4.91 | 297 |

1. Analysis of the mean diffusivity measure (MD)

Voxelwise statistical analysis of mean diffusivity (MD) data was conducted using Tract-Based Spatial Statistics (TBSS) analogically to the FA measure as recommended in the TBSS user guide. MD data were aligned using FNIRT and then transformed into 1 x 1 x 1 mm MNI152 through alignment to the transformed target subject. The transformations were based on the FA analysis, as recommended. Afterward, the MD data was projected onto a mean FA skeleton. Voxelwise cross-subject statistics were performed using randomise.

The group-wise comparison was conducted using permutation testing implemented in randomise, applying Threshold-Free Cluster Enhancement (TFCE). The blind group showed increased MD in numerous white matter tracts compared to sighted controls. Importantly, significant differences were found in the corpus callosum (body and splenium), as well as white matter tracts related to visual processing in the typical population (optic radiation, see Figure S1). Sighted subjects did not show any areas of reduced MD in comparison to the blind subjects.


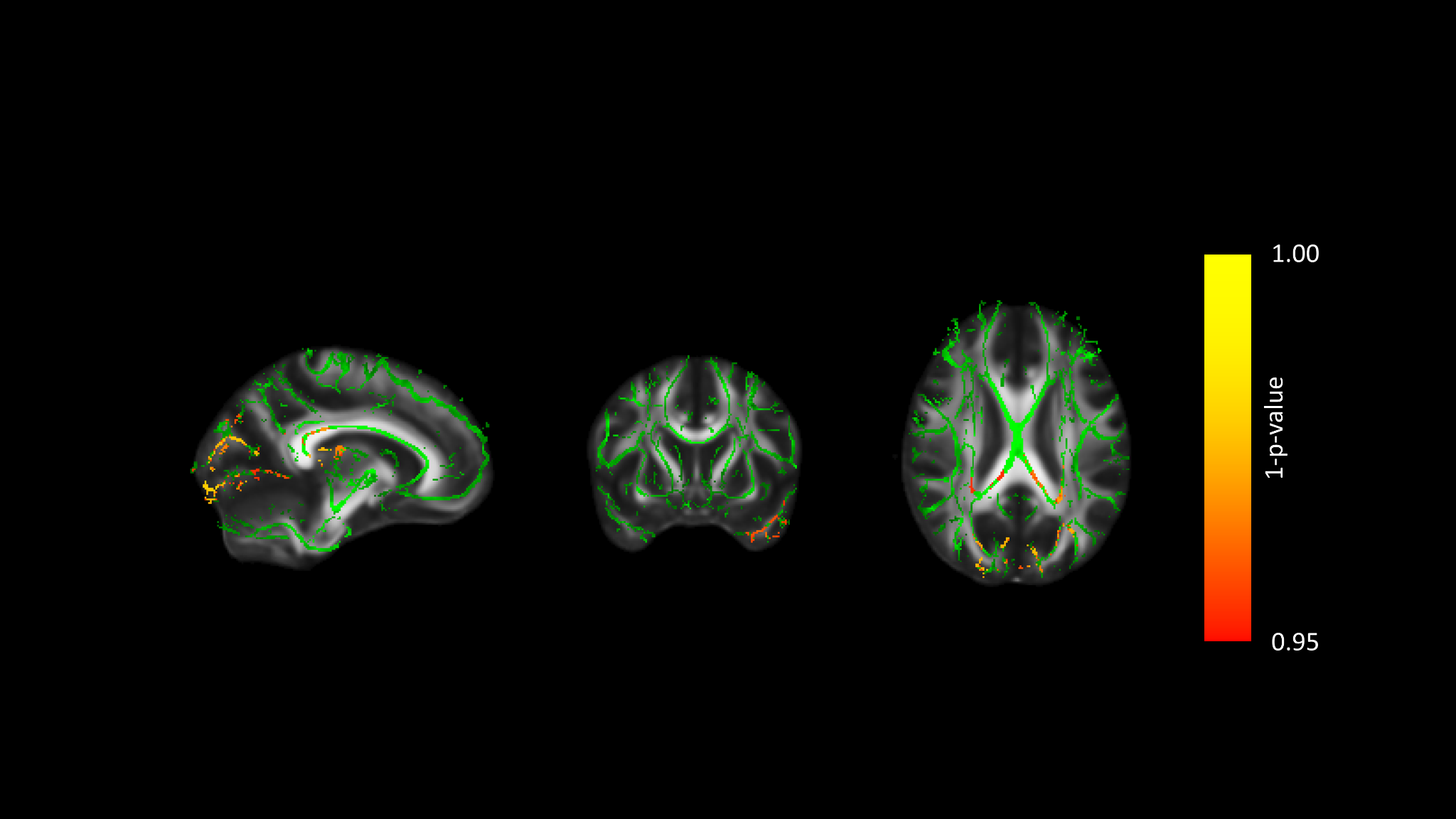


**Supplementary Figure 1.** The mean MD was extracted from the body, genu, and splenium of the corpus callosum (CC), superior longitudinal fasciculus (SLF), and uncinate fasciculus (UF), i.e., from the same ROIs as in the FA analysis. The descriptive statistics of the MD values within groups for the 5 ROIs are presented in Table S3.

**Supplementary Table 4**. MD values (in 10-3 mm2/s) within ROIs for the blind and sighted participants.

|  |  | Blind | Sighted |
| --- | --- | --- | --- |
| CC body | Mean | 0.74 | 0.71 |
|  | SD | 0.1 | 0.03 |
|  | *U* | 385 | |
|  | *p* | 0.162 | |
|  | *r* | 0.2 | |
| CC genu | Mean | 0.65 | 0.64 |
|  | SD | 0.04 | 0.04 |
|  | *U* | 346.5 | |
|  | *p* | 0.516 | |
|  | *r* | 0.09 | |
| CC Splenium | Mean | 0.66 | 0.65 |
|  | SD | 0.05 | 0.03 |
|  | *U* | 364.5 | |
|  | *p* | 0.318 | |
|  | *r* | 0.14 | |
| SLF  asymmetry | Mean | 0 | 0 |
|  | SD | 0.03 | 0.02 |
|  | *U* | 321.5 | |
|  | *p* | 0.99 | |
|  | *r* | < 0.01 | |
| UF asymmetry | Mean | 0 | 0 |
|  | SD | 0.04 | 0.07 |
|  | *U* | 330.5 | |
|  | *p* | 0.734 | |
|  | *r* | 0.05 | |

As the distribution of the MD values was not always normal within the group, U Mann-Whitney’s test was used to compare the group. No significant differences between the groups were found.

Similarly to FA, correlations between the LI within the global language mask and MD values in the ROIs marked in the corpus callosum parts, SLF and UF asymmetry were analysed, controlling for group (blind vs sighted). Similarly to the analysis performed for FA, no significant correlations were found when both groups were analysed together (Table S5). The only significant correlation was observed between the MD values for the genu of corpus callosum and speech processing lateralization in the sighted group. This result is analogical to the results of the analyses on FA values.

**Supplementary Table 5.** Spearman correlation between MD and lateralization of the language network. The correlations for all participants are partial correlations controlled for the blind status. Results surviving Holm-Bonferroni correction are highlighted in bold.

|  |  | Reading: All | Speech: All | Reading: Blind | Speech: Blind | Reading: Sighted | Speech: Sighted |
| --- | --- | --- | --- | --- | --- | --- | --- |
| CC Body | *rho* | 0.09 | 0.10 | 0.02 | 0.03 | 0.30 | 0.21 |
|  | *p* | 1.097 | 1.010 | 0.947 | 1.000 | 0.386 | 0.798 |
| CC Genu | *rho* | -0.25 | 0.24 | -0.28 | -0.03 | 0.20 | **0.58** |
|  | *p* | 0.541 | 0.632 | 0.858 | 1.000 | 0.405 | **0.048** |
| CC Splenium | *rho* | -0.18 | -0.14 | -0.26 | -0.30 | 0.32 | 0.22 |
|  | *p* | 1.023 | 1.090 | 0.766 | 0.785 | 0.495 | 1.000 |
| SLF asymmetry | *rho* | 0.02 | 0.16 | 0.02 | 0.20 | 0.46 | 0.15 |
|  | *p* | 0.877 | 1.200 | 1.000 | 1.000 | 0.217 | 0.553 |
| UF asymmtery | *rho* | 0.15 | 0.05 | -0.31 | -0.01 | 0.44 | 0.34 |
|  | *p* | 1.004 | 0.738 | 0.860 | 0.974 | 0.206 | 0.625 |

1. Analyses without the left-handed participants

## **3.1 Comparison of the lateralization of language processing between blind and sighted**

Two left-handed subjects were excluded from the analyses. The results were largely the same as for the complete sample. The main effects of group (*F*(1, 35) = 6.23, *p* = 0.017) and ROI (*F*(2, 70) = 11.48, *p* < 0.001) were significant, as well as the condition by ROI interaction (*F*(2,70) = 3.23, *p* = 0.046). The main effect of condition (*F*(1, 35) = 0.15, *p* = 0.699), as well as the two-way interaction (group by condition: *F*(1, 35) = 0.89, *p* = 0.355; group by ROI: *F*(2, 70) = 0.67, *p* = 0.516) and three-way group x ROI x condition interaction (*F*(2, 70) = 0.86, *p* = 0.428) were insignificant.

The condition by ROI interaction stemmed from the fact that the ROI effect was significant for both conditions in the sighted group, but only for the speech condition in the blind group. The main effect of ROI indicated that the temporal ROI, in general, had lower LI values (was less left-lateralized) than the frontal and ventral occipitotemporal ROIs (see Supplementary Figure 2). This was not the case for the reading condition in the blind group where all ROIs presented a similar degree of lateralization.


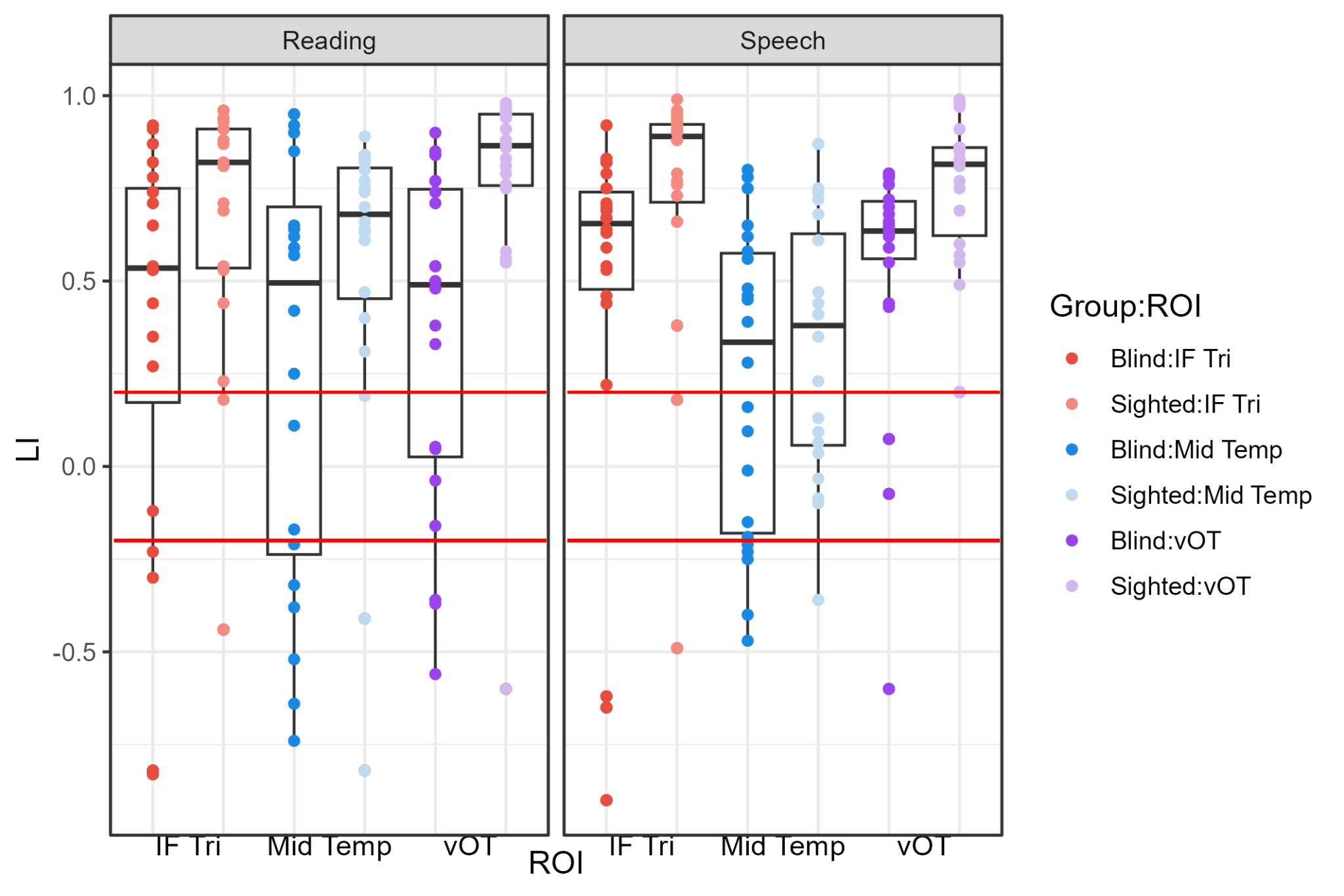


**Supplementary Figure 2.** LI values within groups and ROIs for the reading and speech-processing conditions. The red line marks the LI = 0.2 - a conventional threshold for an activation to be classified as leftward.

The main effect of group indicated that sighted subjects had higher LI values on average than the blind group, indicating stronger left-ward lateralization in this group.

Nevertheless, even in the blind group, the majority of participants presented left-ward lateralization (LI greater than 0.2) for both tasks and all ROIs.

## **3.2 Correlations between the LI and white-matter tracts characteristics**

Similarly to the analyses conducted on the complete sample, none of the correlations turned out to be significant. There were no significant group differences as well.

1. Influence of the reading hand on the lateralization

Lateralization in the language ROIs was compared between the blind participants who use their right hand for reading and those who read with their left hand three-way mixed ANOVA with the reading hand (left vs right) as a between-subjects factor and ROI (IF Tri vs Temp Mid vs vOT) and condition (speech vs reading) as within-subjects factors.

Only the main effects ROI (*F*(2, 38) = 3.19, *p* = 0.052) was approaching significance. The main effect of condition (*F*(1, 19) = 1.39, *p* = 0.254), the main effect of reading hand (*F*(1, 19) = 2.36, *p* = 0.141), as well as all of the interactions (reading hand by condition: *F*(1, 19) = 3.19, *p* = 0.090; condition by ROI: *F*(2, 38) = 0.70, *p* = 0.504; reading hand by ROI: *F*(2, 38) = 0.25, *p* = 0.784, group x ROI x condition (*F*(2, 38) = 0.33, *p* = 0.723)) were insignificant.
